# Ligand based pharmacophore modelling and integrated computational approaches in the quest for small molecule inhibitors against hCA IX†

**DOI:** 10.1039/d3ra08618f

**Published:** 2024-01-22

**Authors:** Venkatesan Saravanan, Bharath Kumar Chagaleti, Shakthi Devi Packiapalavesam, Muthukumaradoss Kathiravan

**Affiliations:** a Department of Pharmaceutical Chemistry, SRM College of Pharmacy, SRM Institute of Science and Technology Kattankulathur Chengalpattu 603203 India; b Dr A. P. J. Abdul Kalam Research Lab, Department of Pharmaceutical Chemistry, SRM College of Pharmacy, SRM Institute of Science and Technology Kattankulathur, Chengalpattu Chennai 603 203 India kathirak@srmist.edu.in drmkkathir@gmail.com

## Abstract

Carbonic anhydrase IX is an important biomarker to fight hypoxic tumours in both initial and metastatic stages of many forms of cancer. Overexpression of hCA IX in the hypoxic environment, has an active role in pH maintenance and makes the hCA IX a better target for the inhibitors targeting specific types of cancer stages. Being a member of the carbonic anhydrase family and having sixteen isoforms, it is important to have a selective inhibition of hCA IX to limit the disruption in the biological and metabolic pathways where other isoforms of hCA are localised and to avoid the other toxicity and adverse effects we try to find selective hCA IX inhibitors from a natural derivative. In the process of finding selective hCA inhibitors we developed a pharmacophore model based on existing inhibitors with IC_50_ values of less than 50 nm, which is then validated with the external decoy set and used for database searching followed by virtual screening to identify the hits based on the pharmacophore fit score and RMSD. Molecular docking studies were performed to identify protein ligand interaction and molecular dynamics simulation studies to analyse the stability of the complex and DFT studies were carried out. The initial screening yielded 43 hits with the RMSD value less than 1, which when subjected to docking exhibited very good interaction with key residues ZN301, HIS94, HIS96 and HIS119. The top 4 compounds in the molecular dynamics simulation studies for 100 ns provided useful insights on the stability of the complex and the DFT studies confirmed the energy variation between HOMO and LUMO is within an acceptable range. An average binding score of −7.8 Kcal mol^−1^ for the lead compounds and high stability margin in the dynamics study concludes that these lead compounds demonstrated outstanding potential for hCA IX inhibitory action theoretically and that further experimental studies for selective inhibition are inevitable.

## Abstract 2

An integrated computational approach in search of potent hCA IX inhibitors.

## Graphical abstract

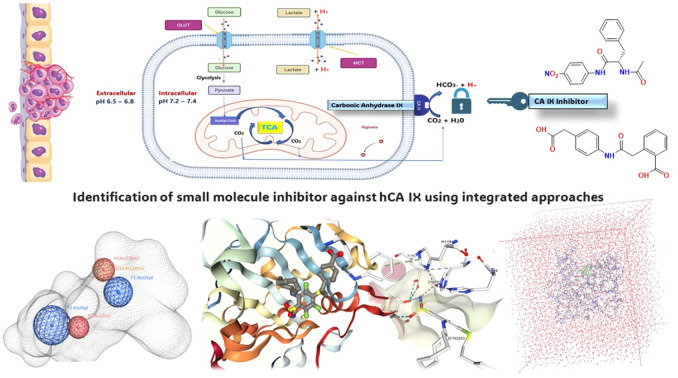

## Introduction

Cancer is the second-greatest cause of death worldwide and a potentially fatal illness. According to estimates, there will be 30 million new instances of cancer by the year 2030.^1^ According to the Cancer Statistics, the United States of America was expected to have 1 958 310 new cases of cancer and 609 820 cancer-related deaths in 2023.^2^ In addition, there is an increase in the prevalence of uterine corpus cancer, prostate cancer, and breast cancer. There are many cancer therapy options and drug combinations available, but they are all associated with severe adverse effects. The rise in side effects and drug resistance to current drugs emphasizes even more the need to bring new, effective therapeutic options to the market.^3^

**Fig. 1:**
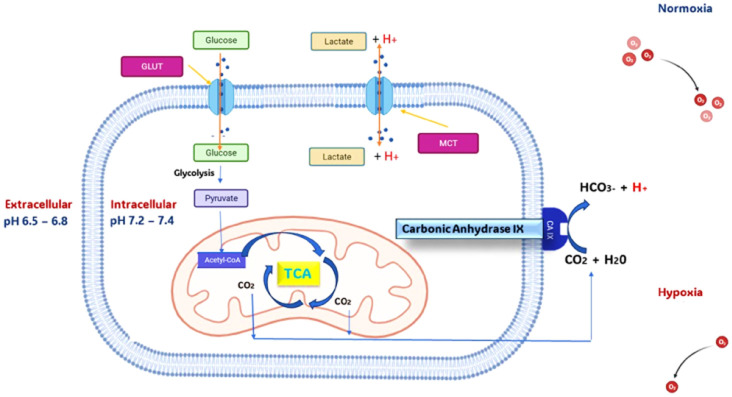
Pictorial representation of carbonic anhydrase IX role in hypoxic tumor cells.

Among the emerging strategies in cancer therapy, targeting human carbonic anhydrases IX and XII (hCA IX and hCA XII) has gained considerable attention because cancer cells are primarily dependent on these enzymes for their survival.^4–6^ The hCA IX and hCA XII are involved in pH regulation and various metabolic processes in hypoxic tumours, in which the vasculature system of tumour mass is unable to meet the oxygen demand of rapidly proliferating cancer cells. Thus, areas with insufficient oxygen supply are generated, which leads to a decrease in ATP production due to reduced oxidative phosphorylation of glucose.^7,8^ This state of tumour hypoxia results in dramatic changes in the gene expression, proliferation, and survival of tumour cells. The hypoxia-triggered metabolic shift towards alternative glycolytic pathways to meet the energy requirements is critical to the survival of dividing cancer cells.^9^Fig. 1 gives insight of hCA IX role in hypoxic tumor cells.

This change also makes the extracellular environment acidic because of the elevated production of lactic as a metabolic end product. This makes the atmosphere very favourable for tumour growth; tumour cells create a hostile environment with the sustained acid availability in the environment, which directly destroys the adjacent host cells. The survival of the cancer cells mainly depends on how well they can adapt to fight the pH change in their environment caused by the accumulated lactic acid and carbon dioxide. This is where the carbonic anhydride IX over-expression helps the cancer cells in this hypoxia condition. The hCA IX in the hypoxic tumour changes the momentum by reducing the pH of the extracellular matrix, which aids the survival and progression of cancer. hCA IX existed and overexpressed in many forms of tumours like breast, colorectal, *etc*, in hypoxic conditions because of its sturdy transcriptional activation favoured by Hypoxia Inductible Factor-1. The existence of hCA IX in non-tumour tissues is rare and generally confined to epithelia of the stomach, gallbladder, pancreas, and intestine. Another issue with tumour hypoxia is that it increases the resistance of the cancer cells towards weakly alkaline chemotherapy medications.^10,11^ Thus, hCA IX an excellent biomarker to tackle the hypoxic tumour in initial and metastatic stages. The involvement of hCA IX in pH regulation and various metabolic processes is crucial, so inhibiting the hCA IX changes the momentum of the hypoxic tumour, suppressing it without any further proliferation.^12^ As hCAs are an extended family of metalloenzymes, the active site contains Zn^2+^, which forms a metal coordination bond with the key residues HIS94, HIS96 and HIS 199, showing itself as a ligand inside the active site, and one hydroxide ion is also present. Various studies claim that to inhibit an hCA, the compound must possess a zinc-binding group. Benzenesulfonamide is one of the hit scaffolds which has been extensively used in the development of human Carbonic Anhydrase Inhibitors (hCAI).^13–17^ This binds with the zinc central metal atom of the protein through a metal coordination bond to cause inhibition. As this is common for all the isoforms of hCAs, every human CA in the α class comprises sixteen isoforms, which vary in tissue distribution, cellular localization, and catalytic activity and range from hCA I to XVI.^18^ While hCA IV, IX, XII, and XIV are attached to the plasma membrane with their catalytic domain facing extracellular space, hCA I–III, VII, and XIII are found in the cytosol. hCA–VA and VB subtypes are limited to the mitochondrial matrix, while hCA VI is secreted in milk and saliva. Only 12 of these hCA isoforms—hCA I-VA, VB-VII, IX, XII, XIII, and XIV—contain Zn(ii) and are catalytically active, while the other isoforms—hCA VIII and X–XI—have no catalytic activity and are also known as CA Related Proteins (CA-RPs).^18–22^ Most of the hCAs are involved in other important metabolic and biological pathways, so inhibiting those isoforms may give rise to various other problems. Hence, designing and developing novel compounds with selective inhibition is important. One such potent hCA IX-selective inhibitor is the 4-(4-fluorophenylureido) benzenesulfonamide (SLC-0111), which is in phase II clinical trials for treating solid hypoxic metastatic tumours.^23^

The hCA IX inhibitors have garnered much attention from researchers in the past decade for developing newer anticancer agents. hCA inhibitors have been used initially as diuretics, antiglaucoma agents, antiepileptics, and altitude sickness management. However, novel generation compounds are undergoing clinical investigation as antiobesity and antitumor drugs/diagnostic tools. None of the therapeutically available hCA inhibitors show any selectivity for a particular isoform.^24,25^ The design and development of isoform-specific hCA inhibitors continue to be difficult task because of the high degree of structural similarity between the hCA isoforms and sequence similarities inside the active area. We have recently reported triazole benzene sulfonamide derivatives^26,27^ as human carbonic anhydrase IX inhibitors using insilico approach like QSAR followed docking and dynamics identified as new lead compound^28–30^ with predicted *K*_i_ = 0.07 nM. Furthermore, we have explored aromatic acid esters in carbonic anhydrase inhibition using CoMFA, CoMSIA and HQSAR^31^ approaches. In continuation to our previous work, we herein developed pharmacophore modelling-based *in silico* analysis as a part of our research to find chemotypes acting as hCA IX inhibitors that could be used in cancer treatment.^32–34^

## Results and discussion

### Ligand-based pharmacophore modelling

**Fig. 2:**
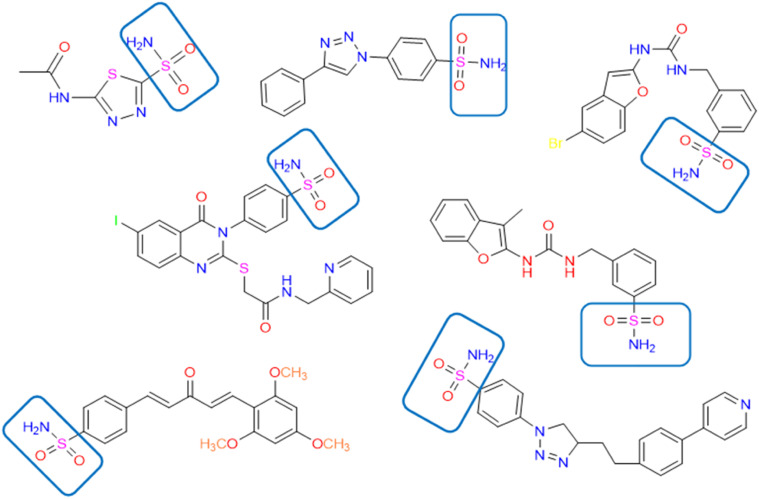
2D structure of the active compounds in the development of the pharmacophore with the zinc binding group.

One of the challenging parts of drug discovery is to tackle the side effects of the compound's non-selective action. The hCA is an extended family of metalloenzymes that plays a key role as a catalyst in converting CO_2_ into carbonic acid. Though more than 16 isoforms of CAs are present, only CA IX and CA XII are over-expressed in cancer, as discussed in the earlier part. So, we aim to find selective CA IX inhibitors without toxicity or adverse effects and hence natural compounds/derivatives similar to the existing CA IX inhibitors might be the key. Hence 7 Chemically active compounds were selected as in Fig. 2 which have proven CA IX inhibition based on the curated literature.

**Fig. 3:**
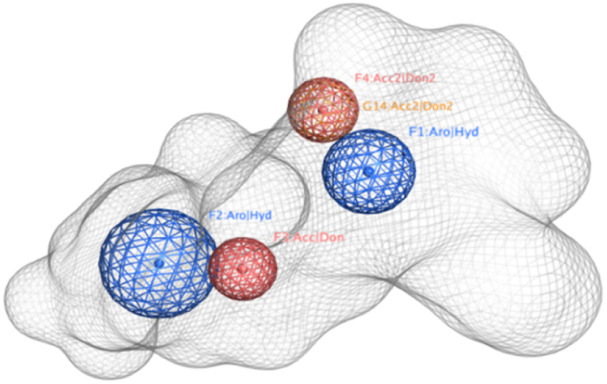
Pictorial representation of the pharmacophore with the selected features generated using the lead compounds.

These 7 compounds were used to develop the pharmacophore model. Pharmacophore query editor interface of molecular operating environment MOE presented us with the features like Aromatic Hydrophobic centers, Hydrogen bond donor acceptor and various other features for the query compound. Different thresholds and their tolerance are tried to enhance the overall pharmacophore model, with values ranging from 50% to 100%. Around 20 hypotheses were created, among which the top model ranged between 86% to 100%. Which is marked as a Ph4.ph4 model with two aromatic hydrophobic centres, F1 and F2 (Aro/Hyd), and two hydrogen bond donor acceptors (F3 and F4: Don/Acc), are shown in Fig. 3. Aromatic hydrophobic interactions are essential for holding a molecule in place within a target protein's hydrophobic binding regions, which increases the stability of the drug–receptor complex. Additionally, the target protein's complementary donor groups can make vital hydrogen bonding connections with all four of the pharmacophoric features. The top model was utilized to construct pharmacophore consensus given in Table 1.

**Table 1:** Pharmacophore consensus with each pharmacophore score, radius, and expression

ID	Score	Radius (A°)	Expression
F1	100%	1.14	Aro/Hyd
F2	86%	1.27	Aro/Hyd
F3	100%	0.66	Don/Acc
F4	86%	1.24	Don/Acc

## Pharmacophore validation

### Pharmacophore validation

The critical ingredient for making the best pharmacophore model is its validation and we tested our model in the pool of active compounds and decoy sets obtained from the DUD-E server. Initial screening with 12 active compounds given in Table S1† and decoys merged was used to run the validation. The true positive signifies the active compounds match the model; the false negative signifies that the active compounds do not match the model; the true negative means the inactive compounds do not match the model, and the false positive means that the inactive compounds match the model. Sensitivity refers to the models' capability to distinguish the active compounds. In contrast, specificity refers to the ability to determine the inactive or decoy compounds and the non-error rate, also known as accuracy or Area under the ROC curve if the data is of binary classification. Simply put, the model with a higher AUC value has better predictability. AUC value ranges from 0 to 1, 1 being the best. So, our model with an average AUC of 0.91 is considered good. This shows the model performance was outstanding in identifying the true actives from the decoys. We triplicated the validation run and took the average to minimize the validation error, and the values are tabulated in Table 2.

**Table 2:** Statistical results of the pharmacophore model validation with triplicated data

S. no	True positive	False negative	True negative	False positive	Sensitivity	Specificity	NER
Trial-1	11	1	487	23	0.91	0.95	0.93
Trial-2	10	2	492	18	0.83	0.96	0.89
Trial-3	11	1	484	26	0.91	0.94	0.92
Average					0.88	0.95	0.91

### Database screening using pharmacophore model

After the validating of pharmacophore model, the 3D query template is subjected to a zinc database to generate lead compounds. The zinc database holds massive numbers of drug-like compounds and reported lead-like compounds along with its information and chemical structure, commercial availability, and biological activity of the compounds. The generated Ph4 pharmacophore model is submitted to the database, and the initial output is lakhs of compounds consisting of natural products, natural derivatives, and synthetic compounds. Then, further filtration is done by keeping the maximum of 0.5 Å RMSD, excluding synthetic compounds from selection; we end up getting 17 524 compounds, which are considered preliminary hits.

### Virtual screening

The generated hits were carried into the virtual screening space for further concentrated results. Then, the pharmacophore model is applied to the 17 524 compounds of natural derivatives, where all the query features were used for screening, and the pharmacophore fit score is the parameter used to identify the hits. The compound with the best score is taken separately, having 43 hits with similar pharmacophore fit and having an RMSD value less than 0.5, which shows that the predicted pharmacophore matches the experimental data. This shows the capability of identifying the structural and functional features responsible for the biological activity of the compounds. These 43 hits exhibited very good pharmacophore fit scores and RMSD values, so they are taken for further investigation.

### Molecular docking studies

#### Active site analysis

**Fig. 4:**
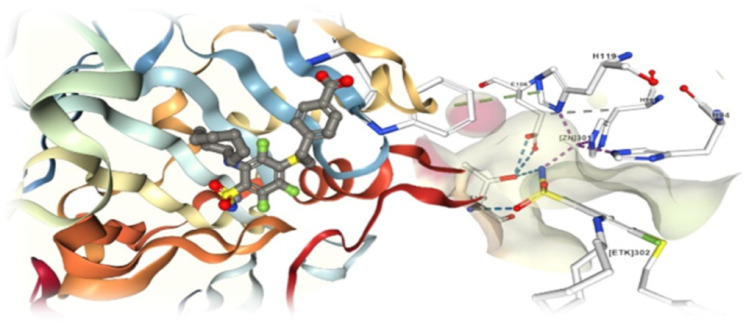
Depicted image showing the active site of the protein with the co-crystallized ligand complex (PDB ID: 6 G9U). The 3D image represented with keeping the residues as a cartoon model and the ligand is represented as ball stick. The 2D interaction diagram on the right shows the key interaction with its residue IDs HIS94, HIS96, HIS119, THR199, ZN301.

Based on the protein structure (PDB), the protein has four chains with sulfonamide as a co-crystallized ligand in all four chains. Hence, all four share a similar binding site and pockets for favourable binding interactions. The active site analysis and binding position were studied to identify the key interactions that can be utilized during molecular docking studies. The analysis of the protein-ligand interactions of all four chains gave us similar results that the ZN301 interaction with the –NH_2_ in the sulfonamide, which is an established Zinc Binding Group (ZBG) and other key interaction includes THR 199 and the proton shuttle residue HIS64, HIS94, HIS96, HIS119. ZN301 is the most emphasized interaction of all, the 3D and 2D interaction is represented in Fig. 4 for better clarity.

#### Molecular docking

Molecular docking is carried out to check the receptor–ligand interaction profile. The selected hits from the pharmacophore-based virtual screening are taken and docked with the protein. As discussed earlier, the protein has four chains that are similar to each other. Standard docking protocol was followed, but only the selected residue in the active site was allowed to get more focused results. This is similar to that of receptor grids in other docking software. The limit was set to generate 30 conformers per ligand and report the best 5 to get the best possible outcome, the docking results of the 43 lead compounds along with the RMSD value and Zinc ID is given in Table S2.† Among the 43 hits, the compounds V3, V18, V19, and V35 showed better binding affinity towards CA IX when compared to that of Acetazolamide with −6.19 K cal^−1^. The binding score and the interacted residues are tabulated in Table 3.

**Table 3:** Zinc ID, binding score, pharmacophore fit score, RMSD and amino acid residues of the top four docked molecule

Compound	Zinc ID	Docking score	Fit score	RMSD	Key residues
V3	Zinc70666499	−7.10 Kcal mol^−1^	95.4	0.950	ZN301, ARG18
V18	Zinc11689965	−7.71 Kcal mol^−1^	95.7	0.704	ZN301, HIS94, THR199,SER197
V19	Zinc72324703	−7.22 Kcal mol^−1^	92.5	0.404	ZN301, HIS94, ARG18
V35	Zinc09419065	−8.05 Kcal mol^−1^	96.3	0.895	ZN301, HIS96, THR199

#### Analysis of protein–ligand interaction

**Fig. 5:**
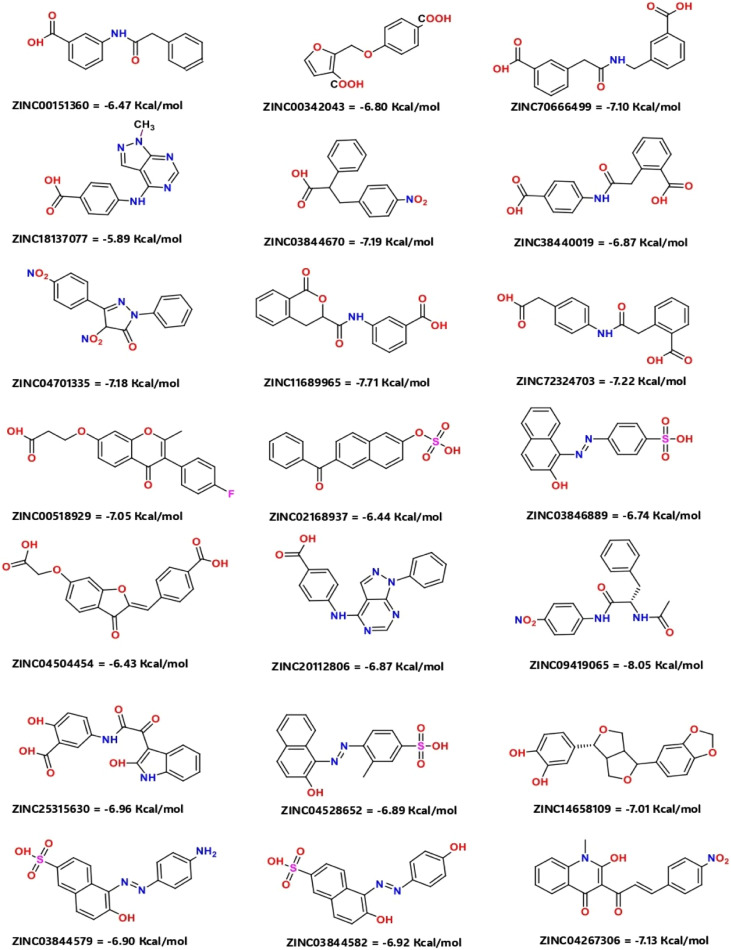
Depicted 2D structure with binding score showing few representative 21 compounds generated from the pharmacophore model based on docking score.

**Fig. 6:**
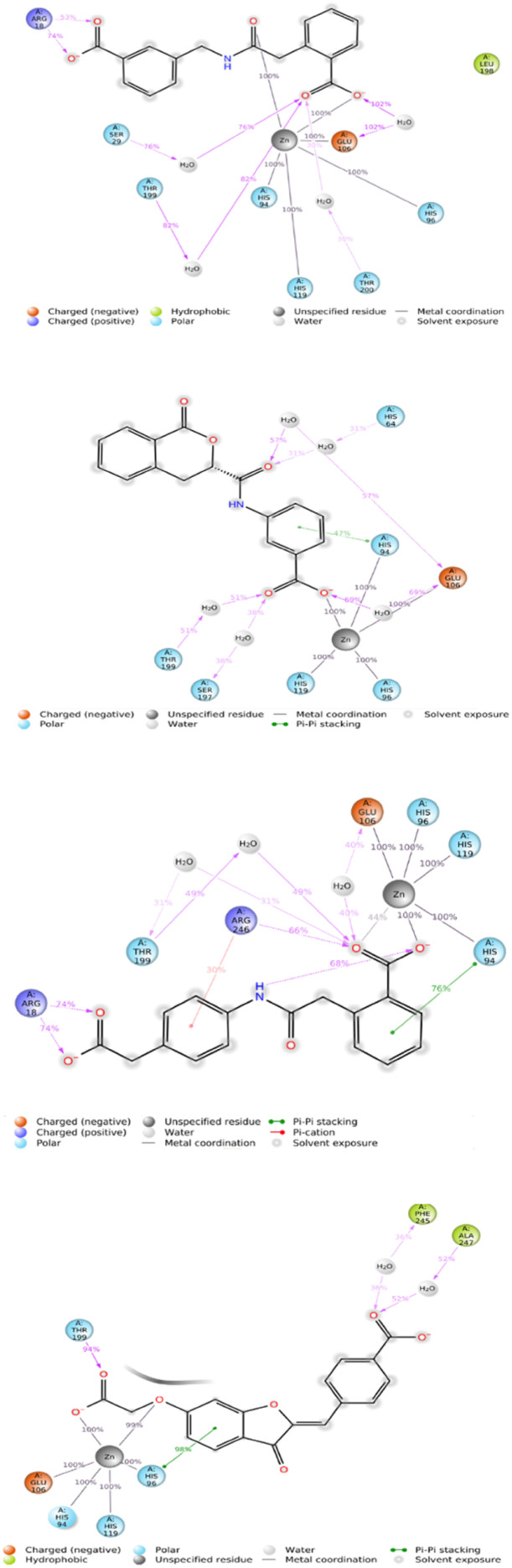
2D interaction diagram of the docked receptor–ligand complex. Here, figures (A) compound V3, (B) compound V18, (C) compound V19, and (D) compound V35 depict the ligand contact with the protein 6G9U. The different color denotes different bonds with the protein and nature of the residues.

**Fig. 7:**
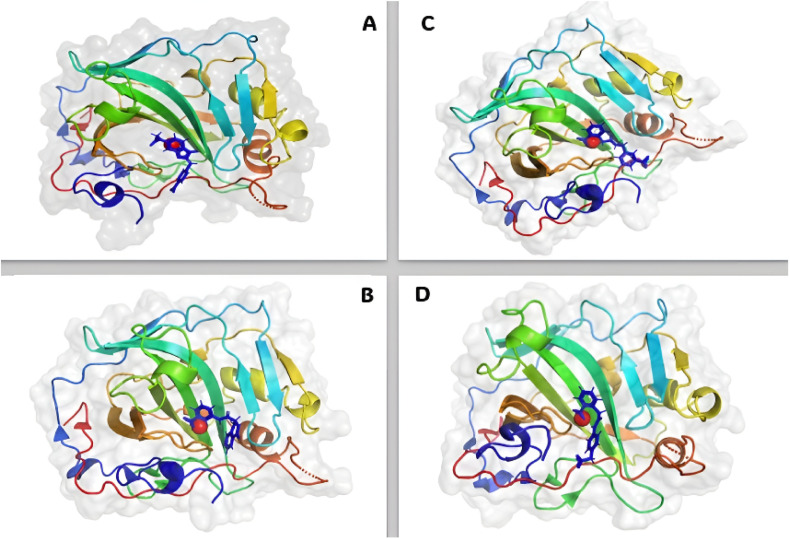
3D interaction diagram of the docked receptor–ligand complex. Here, figures (A) compound V3, (B) compound V18, (C) compound V19, and (D) compound V35 depict the ligand contact with the protein 6G9U.

The molecular docking studies show that the compounds with the best pharmacophore fit score have a better binding affinity towards the receptor. Fig. 5 gives the binding score showing a few representatives of 21 compounds generated from the pharmacophore model based on docking score. Speaking of which compound V3 forms the metal coordination bond with ZN301 in two different positions. As discussed earlier, ZN interaction is the most critical part of designing a CA inhibitor. Zinc comes along with the other three key interaction residues, including HIS 94, HIS 96, and HIS 119; the gatekeeper residues THR 199 are near the ligand; this shows how well the ligand is placed inside the active pocket. The same conditions exist for compound V18, and the metal coordinate bond with ZN 301 and Pi–Pi stacking with HIS 94 and other polar residues SER 197, THR 199, HIS 61 favors the binding. In the case of compound V19, the ZN 301 with metal coordinate bond and its key polar residues HIS 96, HIS 94 and HIS 119, some positively charged ARG 18 favors the interaction in the binding site. Compound V35 shares a similar condition to other ligands with the metal coordinates bonds and hydrophobic interactions at the head, polar residue interactions in the tail, and Pi–Pi stacking with the critical residue HIS 94. Fig. 6 (2D interaction) and Fig. 7 (3D interaction) are the evidence of the same.

#### Pharmacophore mapping

A pharmacophore is a substructure of a molecule which is necessary for a molecule to have a biological activity, a collection of geometrical requirements between particular functional groups that allow the molecule to function biologically. Usually, a pharmacophore model is constructed is two ways, a flexible pharmacophore, and a rigid pharmacophore model. Though the flexibly aligned pharmacophore model takes time to construct and validate, it is said to be the best one for better accuracy. Our 3D model is built on this flexible type. The pharmacophore template used to screen the compounds is then mapped to the hit compounds for further analysis. The hit compounds share the exact pharmacophore fit as the query compound. So, having similar properties can help in getting biological actions identical to those of the query compound.

**Fig. 8:**
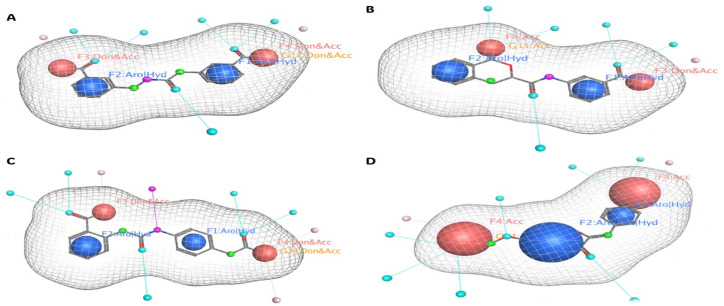
Pharmacophore mapping of the four selected compounds. Here, figures (A) compound V3, (B) compound V18, (C) compound V19, and (D) compound V35. Four pharmacophoric features are represented with the compounds inside the mesh model.

The four pharmacophore features of two aromatic hydrophobic centers (F1 and F2 Aro/Hyd) and two H bond donor–acceptors (F3 and F4: Don/Acc) were mapped into the hit compound, four pharmacophoric features are represented with the compounds inside the mesh model presented in Fig. 8.

### Physiochemical properties analysis and ADMET studies

Any lead/hit compound looks good on paper, but when it is screened for absorption, distribution, metabolism, and excretion, it might or might not clear the test. Critical analysis of ADME properties is much needed; it might cost a fortune for any company if the results go south and fail to meet the criteria. Here, we analyzed our hit compound's ADME properties in the SWISSADME server. The results were interpreted and given in Table 4.

**Table 4:** List of pharmacokinetic properties (physico-chemical, lipophilicity, water solubility, drug likeness, and medicinal chemistry) of the selected compounds

Properties	Parameters	Compound V3	Compound V18	Compound V19	Compound V35
Physico-chemical properties	MW (g mol^−1^)	340.28 g mol^−1^	311.29	313.30	340.28
	Heavy atoms	25	23	23	25
	Arom. heavy atoms	12	12	12	12
	Rotatable bonds	5	4	7	5
	H-bond acceptors	7	5	5	7
	H-bond donors	2	2	3	2
	Molar refractivity	85.85	81.37	83.75	85.85
Lipophilicity	Log *P*_o/w_	1.85	1.38	1.41	1.85
Water solubility	Log *S* (ESOL)	Soluble	Soluble	Soluble	Soluble
Pharmacokinetics	GI absorption	High	High	High	High
Drug likeness	Lipinski, violation	0	0	0	0
Medicinal chemistry	Synth. accessibility	3.10	3.00	2.09	3.10

### Toxicity analysis

Another hurdle in the drug discovery process is drug toxicity. The selected hit compounds are screened for toxicity prediction. Toxicity Estimation Software Tool is an open-source software we used to screen the compounds for Fathead minnow LC_50_, 48 h *Daphnia magna* LC_50_, Developmental toxicity, Oral rat LD_50_ and Bioaccumulation factor. Additionally, Insilico toxicity prediction like ProTox II servers is commonly used due to their high confidence level and accessibility. The preliminary toxicity studies like hepatotoxicity, immunotoxicity, cytotoxicity, carcinogenicity and mutagenicity are calculated using the ProTox II Several toxicological parameters are taken into consideration to get insights into the drug toxicity profile. The values and interpretation are given in Table 5.

**Table 5:** Analysis on the toxicity profile (organ toxicity, toxicity endpoints, androgen receptor, aryl hydrocarbon receptor, heat shock factor response element, phosphoprotein p53, fathead minnow LC_50_ (96 h), developmental toxicity, bioaccumulation factor) of the selected compounds

Endpoint	Target	Compound V3	Compound V18	Compound V19	Compound V35
Organ toxicity	Hepatotoxicity	Inactive	Active (50%)	Inactive	Inactive
Toxicity endpoints	Carcinogenicity	Inactive	Inactive	Inactive	Inactive
	Immunotoxicity	Inactive	Inactive	Inactive	Inactive
	Mutagenicity	Inactive	Inactive	Inactive	Inactive
	Cytotoxicity	Active	Inactive	Inactive	Inactive
	LD_50_ (mg kg^−1^)	700	1000	3000	500
	Toxicity class	4	4	5	4
Tox21-nuclear receptor signaling pathways	Androgen receptor (AR)	Inactive	Inactive	Inactive	Inactive
	Aryl hydrocarbon receptor (AhR)	Inactive	Inactive	Inactive	Inactive
Tox21-stress response pathway	Heat shock factor response element	Inactive	Inactive	Inactive	Inactive
	Phosphoprotein (tumor suppressor) p53	Inactive	Inactive	Inactive	Inactive
Fathead minnow LC_50_ (96 h)	mg L^−1^	NA	2.39	3.20	0.46
48 h *Daphnia magna* LC_50_	mg L^−1^	42.85	52.40	65.60	68.67
Developmental toxicity	Value	0.96	1.05	0.88	0.85
Bioconcentration factor	Log10	0.22	1.44	0.28	0.76

### Molecular dynamics simulation

The molecular dynamics simulation study is used to determine the stability and interaction profile of the complex. The protein–ligand complex of the selected four compounds was subjected to dynamic simulation studies, and the analysis of the complex was performed based on the root mean square deviation, root mean square fluctuation and protein–ligand contacts. The reference time 100 ns simulation of the complex exhibited data positively. The trajectories are analyzed using the simulation diagram interface of the Desmond suite. The trajectory video file of the simulation is given in the ESI.†

#### Relative mean square deviation

In molecular dynamic simulation, the average distance generated by the displacement of the selected atoms over a certain period relative to the reference time frame is measured using the Root Mean Square Deviation (RMSD). RMSD value of the C-alpha and protein fit ligand is computed for all the time frames in the reference time (100 ns).

#### Protein analysis

During the molecular simulation studies, the protein undergoes conformational changes whenever the ligand interacts with the residues. The stability of the complex is much needed for the compound to be suitable for further investigation. Deviation within the range of 1–3 Å is acceptable. If it goes beyond that, the system is declared unstable, and the equilibrium is not attained. In our case, the stability of the complex V3 is within the limit, having a maximum deviation of 2.3 Å, the average RMSD of the protein backbone C-alpha is 1.2 Å, and the complex is stable throughout the simulation time. In the case of complex V18, the average RMSD of 1.5 Å and maximum deviation of 3.0 Å are within the limits, and the system is said to be stable. Complex 19, with an average RMSD of 1.2 Å and a maximum deviation of 2.2 Å, shows a better stability margin for the complex. Complex V35 has a maximum deviation of 2.1 Å, and the stability of the complex is excellent throughout the simulation time, with an average RMSD of 1.2 Å. It possesses the best stability among the four selected hits regarding C-alpha.

#### Ligand analysis

**Fig. 9:**
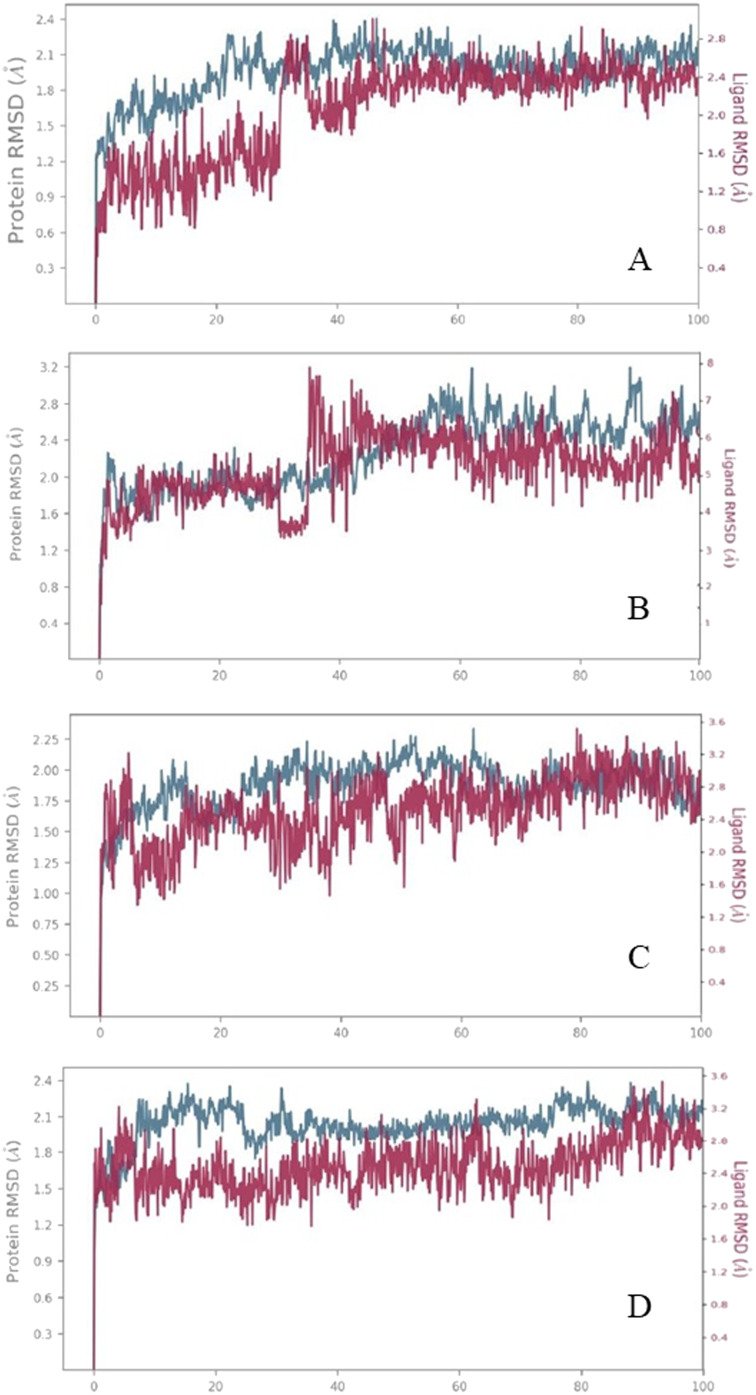
RMSD analysis of the selected compounds. (A) Compound V3, (B) compound V18, (C) compound V19, and (D) compound V35 with respect to reference time of 100 ns.

In the case of ligand fit protein, complex 3 with a deviation of 2.8 Å has some positive and negative deviations during the 40 ns of the simulation time. Complex 18 is the only exception where the RMSD of 5 A is not within the range and showed a high level of instability only regarding ligand fit on protein. Complex 19 with ligand fit protein RMSD of 3.2 Å is within the range, and the stability of the complex is also appreciable. Being the best compound 35 with an average RMSD of 1.6 Å and maximum RMSD of 3.2 Å is within the range, and the complex is stable throughout the simulation time. As the ligand fit protein RMSD is similar to that of protein RMSD, this shows that the ligand doesn't diffuse away from the binding site. It stays on the active site during the entire reference time (100 ns). RMSD of the protein ligand complex in Fig. 9, is the graph to represent the stability of the complex with respect to the reference time of 100 ns. Simulation data for the apoprotein is given in Fig. S1.†

#### Root mean square fluctuation

Root Mean Square Fluctuation (RMSF) in molecular dynamics simulation is used to identify the level of fluctuations the complex undergoes during the conformational changes. Various ligand interaction forces are the contributing factors for this fluctuation. The fluctuation doesn't mean that the complex is unstable. This is because the ligand binding in the receptor makes the complex fluctuate more. Interestingly, the fluctuation of our complex is within the range except in the usual N and C terminals. The secondary structure elements are more rigid than the other parts of the protein.

**Fig. 10:**
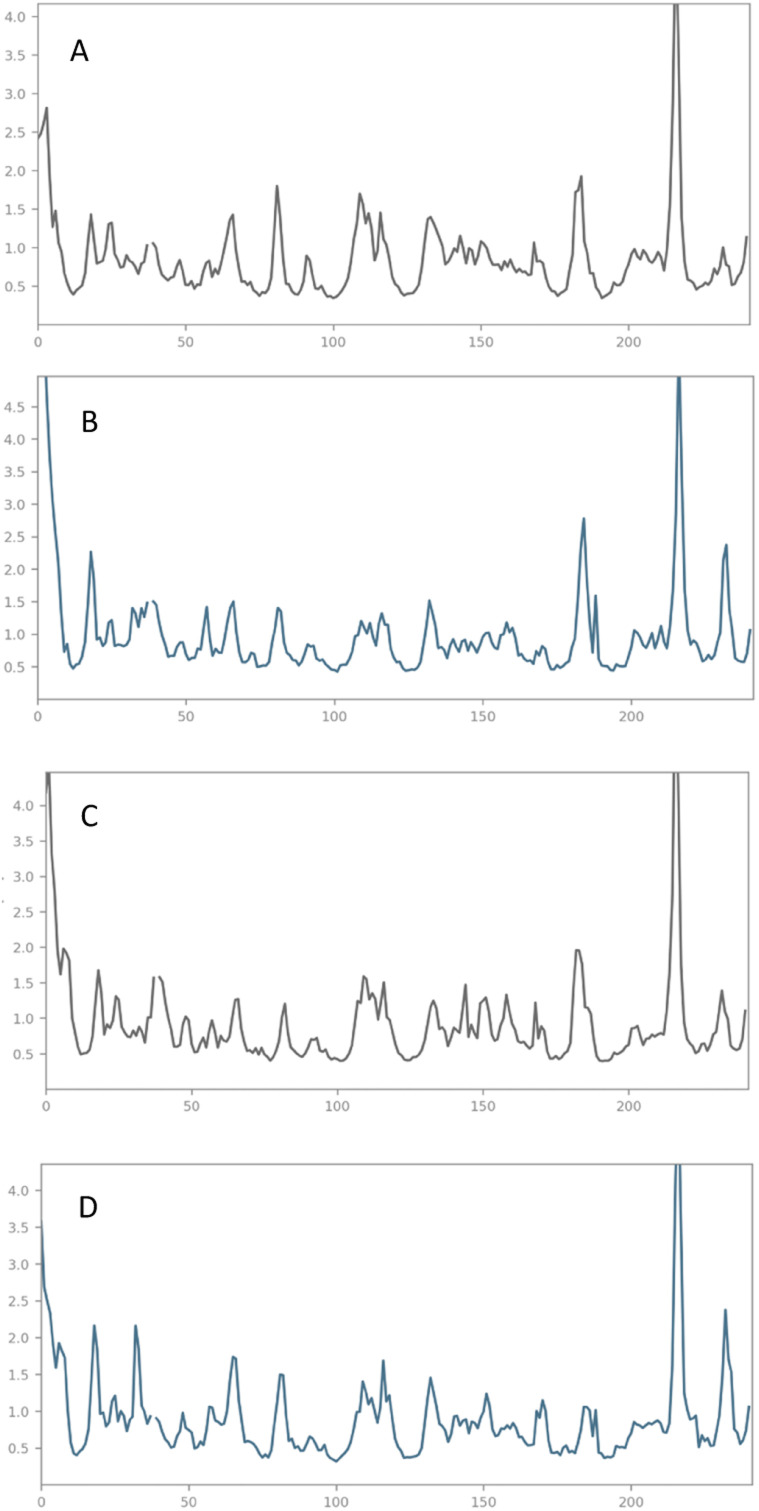
RMSF analysis of the selected compounds. (A) Compound V3, (B) compound V18, (C) compound V19, and (D) compound V35 with respect to reference time of 100 ns.

Hence, the fluctuation is negotiable. The fluctuation throughout the simulation with various residues doesn't creates much fluctuation. The range for the RMSF is also the same less than 3 Å, Compound V3 with average fluctuation less than 2 Å, Compound V18 with fluctuation of 2.5 Å, Compound V19 with less than 1.5 Å and Compound V35 with 2.0 Å stays with the range. Graphical representation of the fluctuation that occurred during the simulation period is given in Fig. 10 for all the lead compounds. All of our hit compounds shared similar RMSF data, which shows our compounds' potential against the receptor.

#### Protein–ligand analysis

**Fig. 11:**
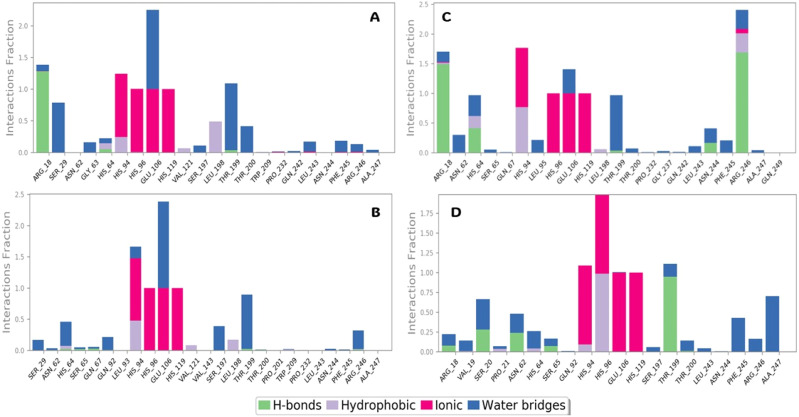
H bond analysis of the selected compounds. (A) Compound V3, (B) compound V18, (C) compound V19, and (D) compound V35 with respect to reference time of 100 ns. Various colors denote different types of interactions with the residues in the *X* axis and the interactions fractions are plotted in *Y* axis.

The interaction of protein and ligand plays a prominent role in designing a molecule to be an effective drug. Ligand interactions are commonly classified into four types: hydrogen bonds, hydrophobic bonds, ionic bonds, and water bridges. While designing a molecule for CA IX, the ionic interaction is the most critical type of binding interaction. Usually, it is H-bonds interaction, but as the CA are part of the metalloenzymes, the ligands commonly interact with ZN, which is favourable with ionic bonds, due to the ionic bond formation with key residues like HIS 94, HIS 96, HIS 119 and GLU 108 is commonly present in all the four screened complexes. H bond interaction with ARG 18 and THR 199 is standard for all and other favourable interaction profiles with water bridges. The fraction in the *Y* axis shows the amount of time the residues interacted with the ligand, having a fraction of 1 means it is in the ligand contact for 100% of the simulation time, so more than 1 fraction indicates that the interaction is even more efficient and sustained for more than 100% of its time. The key residues like HIS94, HIS96, HIS119, GLU106 and THR 199 are in contact with the ligand for more than 1 fraction of time. Various interactions happened during the simulation time is presented in the bar diagram for all the four compounds in Fig. 11.

### Density functional theory

The molecular descriptors were calculated based on the electron density of the compound. Statistical results of the molecular descriptors for compounds V3, V18, V19, and V35 are given in Table 6. The descriptors energy gap, electronegativity, absolute hardness, global softness, chemical potential, and electrophilicity index were calculated based on HOMO and LUMO energy and the charge transfer analysis using Mulliken. The smaller the energy gap, the higher will be the molecular reactivity, meaning they can easily transit from HOMO to LUMO.

**Table 6:** Statistical results of the molecular descriptors for the selected compounds^a^

ID	*E* (RCAM-B3LYP) (in Hartree)	Molecular dipole moment (Debye)	*E*_HOMO_ (in eV)	*E*_LUMO_ (in eV)	Δ*E*	*η*	*σ*	*χ*	*μ*	*ω*	Mulliken (e)
V3	−1081.250	8.79	−0.3089	−0.00760	0.3013	0.15065	3.3189	0.1582	−0.1582	0.0798	0.369
V18	−1080.048	4.79	−0.2885	−0.01843	0.2701	0.13505	3.7023	0.1534	−0.1534	0.0871	0.568
V19	−1081.253	3.66	−0.2597	−0.01577	0.2493	0.12465	4.0112	0.1377	−0.1377	0.0760	0.466
V35	−1212.399	6.38	−0.2903	−0.06129	0.2290	0.11450	4.3668	0.1757	−0.1757	0.1348	0.458

a Δ*E*: energy gap, *η*: absolute hardness, *σ*: global softness, *χ*: electronegativity, *μ*: chemical potential, *ω*: electrophilicity index.

**Fig. 12:**
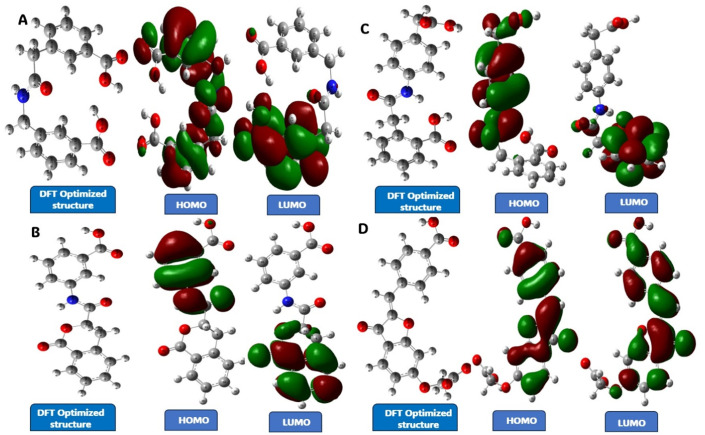
DFT analysis of the selected compounds with its DFT optimized structure with Mulliken charges labelled, HOMO and LUMO. (A) Compound V3, (B) compound V18, (C) compound V19, and (D) compound V35.

The energies are evaluated and represented in Fig. 12. We observed less or similar differences in energy between the HOMO and LUMO for all the compounds, among those four compounds, V35 has the least energy gap of 0.2290. The decrease in electronegativity is proportional to the inhibitory level of the lead compounds. The stronger the electronegativity, the better the inhibitory efficiency. The statistical values of the molecular descriptors are tabulated. Lower chemical hardness and higher global softness values indicates that they are chemically active. The smaller band energy gap, higher dipole moment, and lower electronegativity are vital for the inhibitory effect of a molecule, which was evaluated and validated as having better inhibitory activity of the selected compounds, Mulliken charges used to characterize the charge distribution of the selected compounds. The molecular descriptors calculated during the studies are tabulated in Table 6, and the optimized structure with Mulliken charges label, HOMO and LUMO positions of the lead compounds is given in Fig. 12.

## Methodology

### Pharmacophore model generation

**Fig. 13:**
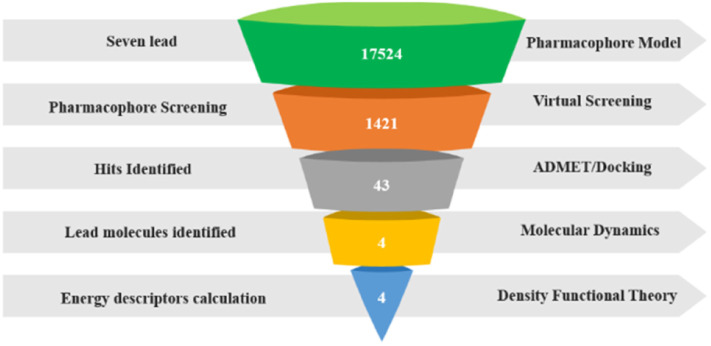
Overall workflow and methodology.

To construct any good ligand-based pharmacophore model, it is important to have best lead compounds curated by conducting a extensive literature review. The 7 potent active lead obtained from various literature are downloaded from the PubChem Database in SDF format. Then it is taken to the Molecular Operating Environment (MOE), structures are energy minimized using CHARMM forcefield to develop the pharmacophore model. As per reported procedure the pharmacophore model was built. The pharmacophore query editor interface of MOE^35^ interprets various pharmacophoric features, it is then examined carefully and excluded or included features whichever is essential to construct the best pharmacophore model. The overall workflow and the methodology is given in Fig. 13. We constructed around 20 hypotheses after refinement using various filters and the best model is identified based on the least negative score (S) and Standard Deviation (SD). It is taken for further steps.^28^

#### Pharmacophore validation

Validation is essential to evaluate the confidence level of the pharmacophore model in distinguishing active and decoys. The database consists of 12 active lead compounds and decoys downloaded from the DUD-E servers is created and taken to the MOE system. Then, the pharmacophore model is subjected in that database for validation. The pharmacophore model was validated based on three parameters: Sensitivity (Sn), specificity (Sp), and non-error rate (NER).^36^ These parameters can be calculated using the below equation:
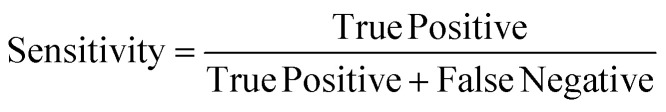

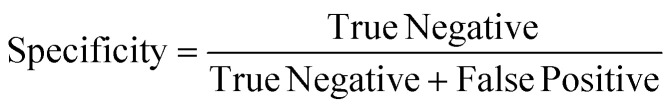

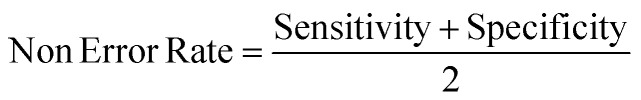


### Database curation and virtual screening

Once the validation is accomplished, the pharmacophore is taken for further database screening. Zinc database^37,38^ is an open-source chemical library having more than 37 billion compounds, it is used to generate the hits compounds by using the developed pharmacophore as 3D query template saved as .Ph4 format. The preliminary screening with standard protocol yields 17 524 compounds, then based on our requirements various filters are used. The conditions were used for filtrations are (i) Lipinski's rule of five (ii) Root Mean Square Deviation (RMSD) less than 0.5 A. (iii) Screening restricted to natural derivatives. After the filtration based on the RMSD and pharmacophore fit, top 43 hits are taken for further processing using molecular docking studies.

### Physicochemical properties analysis and ADME studies

Drug likeness analysis is one of the crucial steps during drug discovery because many of the lead like compounds fails in clinical trials due to poor ADME properties and significant toxicity effects on biological systems. *In silico* predictions are helpful in designing novel compounds prior to the preclinical and clinical trials, thus save time and costs. The biological effects of the drugs are mainly dependent upon its physicochemical properties. To calculate the ADME properties, we have used SWISS ADME server,^39^ which is also an open access tool to predict the ADME properties, the procedure to calculate is by converting the compounds in SMILES format and pasting the SMILES in the tool gives direct results, we picked the focused data and interpreted.

### Toxicity analysis

It is now feasible to test toxicity using *in silico* methods to obtain the necessary chemicals' safety profile through computationally based approaches. The toxicity profile has the ability to assess and ascertain the immunotoxicity, LD_50_ value, carcinogenicity, and mutagenicity in both quantitative and qualitative ways. As like physiochemical properties calculation, toxicity prediction using ProTox II server^40^ is very much straightforward. We can upload the compounds in Sdf format or draw using the inbuilt tool or by pasting the SMILES, the server generates the toxicity results in less than 10 seconds. Another software used to calculate the toxicity profile is Toxicity Estimation Software Tool^41^ is an open-source software we used to screen the compounds for Fathead minnow LC_50_, 48 h *Daphnia magna* LC_50_, developmental toxicity, oral rat LD_50_ and bioaccumulation factor.

### Molecular docking

A useful technique for lead optimization and discovery is molecular docking.^42^ Numerous search techniques and grading systems have been used in the development of docking programmes over the last thirty years. A computer programme called “molecular docking” finds every potential interaction that could occur between the ligand and the target active site. After being identified as hit compounds by the Pharmacophore model, MOE® 2022 (ref. 35) molecular docking was used for further screening with CA XII as the target. Molecular docking was achieved using the rigid receptor docking approach, wherein the CA IX was configured to be rigid, and the ligands were set to be flexible. Using MMFF94x force field settings, precise molecular simulations and structural studies within MOE were obtained.

The carbonic anhydrase active site is a conical cavity with hydrophobic and hydrophilic sections, with the Zn(ii) ion located at the bottom. The X-ray crystal structures of the CA IXII (PDB ID 6G9U) were obtained from the Protein Data Bank. Our goal was to determine if the filtered hit compounds stick to the active site with the necessary shape in a similar way to what was shown with Co crystallized ligands. The downloaded PDB file underwent an energy minimization technique before the docking operation began. The selected Proteins has four chains, A, B, C and D. Protein without water molecules and co-crystallized ligands except zinc were separated. There are twenty-five active sites in the 6G9U protein. The active site's size and interactions with the co-crystallized residues play a major role in the selection process. MOE received and prepared the 43 hits from the ZINC database, then energy minimization was carried out. The MOE docking methodology was used to model the molecular docking of natural ligands to the target protein. The docking procedure was executed after the scoring function and placement scoring were merged. 3D figures were created and visualized using the MOE® 2022 programme.

### Molecular dynamics simulation

Molecular dynamics simulation was carried out with Desmond V 5.9 Package Schrodinger LLC suite in Ubuntu environment. It is part where the protein's stability of the complex is tested. Massive amount of computing power is needed to simulate for 1 second, so 100 ns of data is universally accepted data. We built our computing workstation with 16 Gigabytes of Nvidia GTX3060 graphic card serves as a GPU, the CUDA platform is installed to sync the GPU with the Desmond environment. The best possible conformer is saved as a PDB complex for dynamics was then energy minimised with an OPLS forcefield. The position of the complex was centred in an orthorhombic cubic box, with TIP3P water molecules as per the standard protocols, in addition to the buffers of nearly 10 Angstrom between the protein atom and the box edge for dynamics simulation. In order to neutralize the system, the box volume has been set based on the complex and counterions such as Na^+^ and Cl^−^ ions. Using the OPLS-2005 force field parameter, minimization was carried out in accordance with the Desmond protocol, and the NPT and NVT ensemble was kept in place to maintain the temperature at 10 K in order to constrain heavy atoms on the solute. The simulation was run using a relaxation time of 20 ps, one atmospheric pressure, and a temperature of roughly 300 K. The NPT parameters are kept standard and the simulation reference time of 100 ns was initiated. The simulation diagram interface of Desmond is used to analyze the trajectory.^43,44^

### Density functional theory

Density functional theory is a quantum mechanical technique to describe the small molecular structural characteristic and to understand the chemical behaviour utilizing the energy in their molecular orbital. It provides the understanding of the relationship between the biological activity of the compound and their electronic properties.^45^ To commence, the compounds were optimized using Heyd–Scuseria–Ernzerhof hybrid functional exchange – correlation functional named CAM-B3LYP function and 6-31G(d) basis set in Gaussian 16 software. Total energy, dipole moment, highest occupied molecular orbital (HOMO) and lowest unoccupied molecular orbital (LUMO), band energy gap (Δ*E*), electronegativity (*χ*), chemical potential (*μ*), global softness (*σ*), absolute hardness (*ω*), electrophilicity index (*ω*) and Mulliken charges were the descriptors calculated for the chosen compounds. These descriptors influence the interaction of the ligand molecule in the binding pocket of the protein.^46–48^

## Conclusion

The generated ligand-based pharmacophore model was found to be good with top model Ph4.ph4 ranged between 86% to 100% with an average AUC of 0.91% obtained from the pharmacophore validation were triplicated showing the pharmacophore models confidence. The hits derived from the virtual screening is subjected to docking produced good binding interactions (PDB ID: 6G9U) with score of compound V3 (ZINC70666499) = −7.10 Kcal mol^−1^, V18 (ZINC11689965) = −7.71 Kcal mol^−1^, V19 (ZINC72324703) = −7.22 Kcal mol^−1^ and V35 (ZINC09419065) = −8.05 Kcal mol^−1^ against the standard Acetazolamide −6.19 Kcal mol^−1^. Metal coordination bond formation with key residues like ZN301, HIS94, HIS96 and HIS119 shows the presence of potential ZBG in the lead compound. The physiochemical studies of the natural derivatives has a better chance of being a CA IX inhibitors with zero Lipinski rule of five violation and toxicity profile shows the lead compounds are cleared the various toxicity parameters. Molecular dynamics simulation reported that the compounds V3, V19, and V35 exhibited very good and similar stability margin, all the values are under the deviation limit of 3 A, except V18 with the deviation of 5 Å in the ligand RMSD, although all other values of V18 are within the range. The DFT study's findings are that energy gap, higher dipole moment, lower electronegativity and Mulliken charges are in favor of lead compounds which vital for the inhibitory effect of a molecule. Based on our results, the three lead compounds compound V3 (ZINC70666499), V19 (ZINC72324703) and V35 (ZINC09419065) demonstrated outstanding potential for hCA IX inhibitory action theoretically and that further experimental studies for selective inhibition are inevitable.

## Author contributions

VS: data collection, conceptualization, data curation, investigation, methodology, validation, writing-original draft. BK: writing & editing, MK: resources, software, supervision, writing review & editing.

## Conflicts of interest

There are no conflicts to declare.

## Supplementary Material

RA-014-D3RA08618F-s001

RA-014-D3RA08618F-s002
