# Supplementary material for: Ligand based pharmacophore modelling and integrated computational approaches in the quest for small molecule inhibitors against hCA IX
Source: RSC Adv. 2024 Jan 22;14(5):3346–58. doi: 10.1039/d3ra08618f (PMC10801456; doi:10.1039/d3ra08618f)
Supplement: RA-014-D3RA08618F-s002 [file RA-014-D3RA08618F-s002.pdf]

Electronic Supplementary Material (ESI) for RSC Advances.
This journal is © The Royal Society of Chemistry 2024

Ligand based pharmacophore modelling and integrated computational approaches in the quest of small molecule inhibitor against hCA IX

S.No	SMILES

Ligand based pharmacophore modelling and integrated computational approaches in the quest of small molecule inhibitor against hCA IX

Ligand ID	ZINC ID	Docking score	RMSD	Amino acid interaction
V1	Zinc00151360	-6.47	0.841	HIS 94, ZN 301
V2	Zinc00342043	-6.80	0.916	THR 199, ASN 62, SER 65, ZN 301
V3	Zinc70666499	-7.10	0.950	ARG 18, ZN 301

1	CC(NC1=NN=C(S(=O)(N)=O)S1)=O
[illegible]	[illegible]
3	O=C(/C=C/C1=C(OC)C=C(OC)C=C1OC)/C=C/C2=CC=C(S(N)(=O)=O)C=C2
4	O=S(C(C=C1)=CC=C1N(N=N2)CC2CCC(C=C3)=CC=C3C4=CC=NC=C4)(N)=O
5	O=S(C1=CC=C(N2C=C(C3=CC=CC=C3)N=N2)C=C1)(N)=O
6	CC1=C(NC(NCC2=CC(S(=O)(N)=O)=CC=C2)=O)OC3=CC=CC=C13
7	O=S(C1=CC=CC(CNC(NC2=CC3=CC(Br)=CC=C3O2)=O)=C1)(N)=O
8	NS(C1=CC=C(NC(NC2=NC(C)=C(C)N=C2C)=O)C=C1)(=O)=O
9	CN1C[C@@H]2[C@@H](C[C@@H]([C@]2(C(=O)O)NC(=O)CS(=O)(=O)N)O)C(=C1)C(=O)N
10	C1=CC(=C(C=C1C/C(=N\O)/C(=O)NCCS(=O)(=O)N)Br)O
11	O=C([Se]CC#C)CC(C1=C2C=CC(OC)=C1)=C(C)N2C(C3=CC=C(Cl)C=C3)=O
12	FC(F)(F)C1=CC=CC(NC2=C(C([Se]CC#C)=O)C=CC=C2)=C1

Table S1. Active Compounds used in the pharmacophore validation.

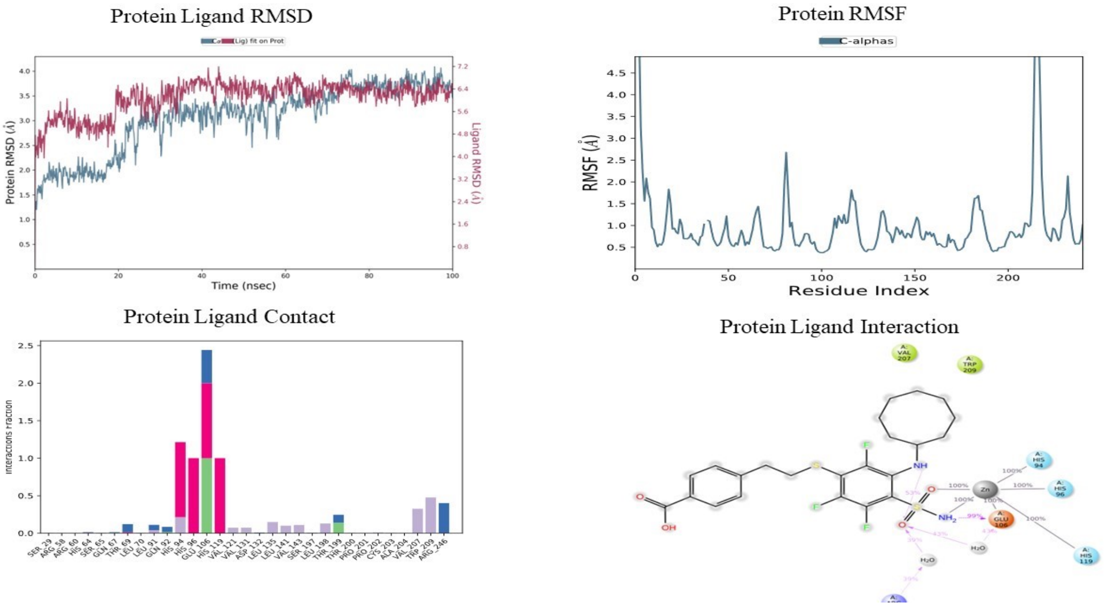

Figure S1. Molecular dynamics simulation data of the Apoprotein.

**V4**	Zinc02420638	-5.97	0.761	THR 199, HIS 94
**V5**	Zinc18137077	-5.89	0.929	GLN 92, ZN 301
**V6**	Zinc01767896	-5.80	0.593	ASN 62, GLN 92
**V7**	Zinc03844670	-7.19	0.881	THR 199, HIS 94, ZN 301
**V8**	Zinc13532309	-5.58	0.957	VAL 121, ZN 301
**V9**	Zinc03845641	-6.09	0.606	GLN 92, ASN 62
**V10**	Zinc01704331	-5.60	0.957	HIS 94, ZN 301
**V11**	Zinc00536677	-6.11	0.906	ASN 62, HIS 94
**V12**	Zinc01505327	-6.38	0.770	HIS 94
**V13**	Zinc00003955	-7.08	0.835	ZN 301
**V14**	Zinc38440019	-6.87	0.818	GLN 92, GLN 67, ZN 301
**V15**	Zinc04994155	-6.03	0.936	HIS 96, ZN 301
**V16**	Zinc0049817	-6.28	0.886	HIS 94, ZN 301
**V17**	Zinc04701335	-7.18	0.631	THR 199, ZN 301
**V18**	Zinc11689965	-7.71	0.704	HIS 94, THR 199, SER 197, ZN 301
**V19**	Zinc72324703	-7.22	0.404	THR 199, ARG 60, ZN 301
**V20**	Zinc04180215	-5.65	0.498	HIS 94, ARG 18, ZN 301
**V21**	Zinc68572305	-6.04	0.917	ASN 62, ZN 301
**V22**	Zinc19703228	-6.57	0.830	VAL 121, ZN 301
**V23**	Zinc19732669	-5.96	0.954	HIS 94, GLN 92, ASN 62
**V24**	Zinc03847982	-5.85	0.920	GLN 92, ZN 301
**V25**	Zinc12620605	-6.34	0.956	THR 199, GLN 92, ASN 62
**V26**	Zinc00518929	-7.05	0.795	LEU 198, THR 199, ZN 301
**V27**	Zinc02168937	-6.44	0.576	LEU 91, THR 199, ZN 301
**V28**	Zinc03846889	-6.74	0.933	THR 199, ZN 301
**V29**	Zinc04261842	-6.64	0.769	THR 199, HIS 94, ZN 301
**V30**	Zinc04504454	-6.43	0.764	HIS 94, ZN 301
**V31**	Zinc01675744	-6.39	0.855	ASN 62
**V32**	Zinc20112806	-6.87	0.967	GLN 92, ZN 301
**V33**	Zinc13544730	-6.64	0.551	THR 199, ZN 301
**V34**	Zinc13406180	-8.07	0.737	GLN 92, ZN 301
**V35**	Zinc09419065	-8.05	0.895	ZN 301, HIS 96, THR 199
**V36**	Zinc25315630	-6.96	0.567	ZN 301, HIS 94
**V37**	Zinc04528652	-6.89	0.922	THR 199, ZN 301
**V38**	Zinc68581601	-6.06	0.494	ASN 62, THR 199, ZN 301
**V39**	Zinc14658109	-7.01	0.796	HIS 94
**V40**	Zinc03844579	-6.90	0.769	HIS 94, VAL 121, ZN 301
**V41**	Zinc03844582	-6.92	0.767	THR 199, THR 69, ZN 301
**V42**	Zinc70666213	-6.40	0.950	ASN 244, HIS 94
**V43**	Zinc04267306	-7.13	0.928	HIS 94, ZN 301

**Standard**	Acetazolamide	-5.96		HIS 94, ASN 62, THR 200, ZN 301

Table S2. Lead compounds generated from Zinc database with its ZINC ID, RMSD value, Binding score, and Amino acid residue.
